# The Impact of Serum Albumin Levels on COVID-19 Mortality

**DOI:** 10.3390/idr14030034

**Published:** 2022-04-20

**Authors:** Verena Zerbato, Gianfranco Sanson, Marina De Luca, Stefano Di Bella, Alessandra di Masi, Pietro Caironi, Bruna Marini, Rudy Ippodrino, Roberto Luzzati

**Affiliations:** 1 Infectious Diseases Unit, Trieste University Hospital (ASUGI), 34125 Trieste, Italy; verena.zerbato@gmail.com; 2 Clinical Department of Medical, Surgical and Health Sciences, Trieste University, 34149 Trieste, Italy; gianfranco.sanson@gmail.com (G.S.); roberto.luzzati@asugi.sanita.fvg.it (R.L.); 3 Operative Unit of Medicina Clinica, Trieste University Hospital (ASUGI), 34125 Trieste, Italy; marinadeluca87@gmail.com; 4 Department of Sciences, Roma Tre University, 00146 Roma, Italy; alessandra.dimasi@uniroma3.it; 5 Department of Anaesthesia and Critical Care, AOU S. Luigi Gonzaga, Department of Oncology, University of Turin, 10043 Turin, Italy; pietro.caironi@unito.it; 6 Ulisse BioMed Labs, Area Science Park, SS 14, km 163.5, 34149 Trieste, Italy; b.marini@ulissebiomed.com (B.M.); r.ippodrino@ulissebiomed.com (R.I.)

**Keywords:** serum albumin, hypoalbuminemia, SARS-CoV-2, COVID-19, mortality, invasive mechanical ventilation, length of hospital stay, respiratory failure

## Abstract

Low serum albumin (SA) correlates with mortality in critically ill patients, including those with COVID-19. We aimed to identify SA thresholds to predict the risk of longer hospital stay, severe respiratory failure, and death in hospitalized adult patients with COVID-19 pneumonia. A prospective longitudinal study was conducted at the Infectious Diseases Unit of Trieste University Hospital (Italy) between March 2020 and June 2021. The evaluated outcomes were: (1) need of invasive mechanical ventilation (IMV); (2) length of hospital stay (LOS); and (3) 90-day mortality rate. We enrolled 864 patients. Hypoalbuminemia (<3.5 g/dL) was detected in 586 patients (67.8%). SA on admission was significantly lower in patients who underwent IMV (2.9 vs. 3.4 g/dL; *p* < 0.001). The optimal SA cutoff predicting the need of IMV was 3.17 g/dL (AUC 0.688; 95% CI: 0.618–0.759; *p* < 0.001) and this threshold appeared as an independent risk factor for the risk of IMV in multivariate Cox regression analysis. The median LOS was 12 days and a higher SA was predictive for a shorter LOS (*p* < 0.001). The overall 90-day mortality rate was 15%. SA was significantly lower in patients who died within 90 days from hospital admission (3.1 g/dL; IQR 2.8–3.4; *p* < 0.001) as compared to those who survived (3.4 g/dL; IQR 3.1–3.7). The optimal SA threshold predicting high risk of 90-day mortality was 3.23 g/dL (AUC 0.678; 95% CI: 0.629–0.734; *p* < 0.001). In a multivariate Cox regression analysis, SA of <3.23 g/dL appeared to be an independent risk factor for 90-day mortality. Our results suggest that low SA on admission may identify patients with COVID-19 pneumonia at higher risk of severe respiratory failure, death, and longer LOS. Clinicians could consider 3.2 g/dL as a prognostic threshold for both IMV and mortality in hospitalized COVID-19 patients.

## 1. Introduction

On 11 March 2020, World Health Organization (WHO) declared Coronavirus Disease 19 (COVID-19) a pandemic [1]. COVID-19 is caused by severe acute respiratory syndrome coronavirus 2 (SARS-CoV-2), a positive-sense single-stranded RNA virus, member of *Coronaviridae* family, genera *Betacoronavirus*, first identified on 9 January 2020 in Wuhan, China [2]. Most patients with COVID-19 have mild disease with fever, dry cough, and fatigue. Many infections are asymptomatic, particularly in children and young adults. Older people and/or people with co-morbidities are at higher risk of severe pneumonia, respiratory failure, and death [2]. Since the beginning of the pandemic, scientists’ efforts were focused on identifying COVID-19 risks and prognostic factors [3]. In critical illness, such as sepsis or trauma, low serum albumin (SA) concentrations have been associated with poor outcomes [4,5].

Albumin is the most abundant circulating protein in the plasma. It is synthesized by hepatocytes and rapidly excreted into the bloodstream, where it carries out several functions including the modulation of plasma oncotic pressure and the transport of endogenous and exogenous ligands such as drugs [6,7]. Hypoalbuminemia may be the result of decreased production of SA (for example in cirrhosis), of increased loss of SA via the kidneys, gastrointestinal tract, skin, or extravascular space (for example in acute and chronic inflammation), of increased catabolism of SA, or, more often, a combination of these mechanisms [8]. The severity of hypoalbuminemia reflects the severity of both acute and chronic inflammation. Moreover, hypoalbuminemia is associated with the acquisition and severity of infectious diseases [9].

The association between SA levels and disease severity and mortality has been investigated also in COVID-19 patients. Huang et al., for the first time, found out that hypoalbuminemia may be an independent predictor of mortality in COVID-19 hospitalized patients [10]. Similarly, Kheir et al., in a retrospective study including hospitalized patients with SARS-CoV-2 pneumonia, observed that higher albumin levels on admission were associated with significantly fewer adverse outcomes, including venous thromboembolism, the development of acute respiratory distress syndrome, intensive care unit (ICU) admissions, and readmissions within 90 days [11]. Recently, a meta-analysis of Soetedjo et al. confirmed the association between hypoalbuminemia and poor prognosis in COVID-19 patients [12].

The aim of our study was to identify, through a cohort prospective observational study, SA thresholds to predict the need of invasive mechanical ventilation (IMV), the risk of longer hospital stay, and the risk of death in hospitalized adult patients with COVID-19 pneumonia.

## 2. Materials and Methods

### 2.1. Study Design and Population

A prospective longitudinal cohort study was carried out at Trieste University hospital, Italy. All adult patients (aged > 18 years) consecutively admitted to the Infectious Diseases Unit from March 2020 to June 2021 were considered for inclusion if affected by COVID-19 pneumonia. Diagnosis of COVID-19 pneumonia included a Nucleic Acid Amplification Test positive for SARS-CoV-2 and radiographic findings suggestive of pneumonia. Patients without SA determination at hospital admission and those who refused to participate were excluded from the study.

### 2.2. Data Collection

Demographics and clinical data were collected on admission. Hypertension, heart disease, diabetes, obesity, and metastatic cancer were considered as potential risk factors for a poor prognosis [13]. A body mass index (BMI) threshold of ≥30 kg/m^2^ was selected to identify subjects with obesity.

The following laboratory tests were performed on hospital admission: C-reactive protein (CRP), D-dimer, white blood cell count, and SA.

Hypoalbuminemia was defined as a SA < 3.5 g/dL, based on the hospital laboratory indications.

### 2.3. Study Endpoints

The performance of SA in predicting subjects at risk of having a poor outcome was investigated in order to identify the SA cut off able to better predict a patient’s prognosis since hospital admission. Moreover, the predictive power of the identified SA thresholds was tested via multivariable models including relevant variables independently associated with each explored outcome.

### 2.4. Study Outcomes

Patients were followed up to 90 days after hospital admission. The evaluated outcomes were: (1) need of IMV; (2) hospital length of stay (LOS); and (3) 90-day mortality rate. The need for IMV was considered as a proxy of the most severe respiratory condition. Since the decision to intubate a patient may have been influenced by contingent conditions, such as the limitation of the available resources (e.g., ICU beds or ventilators) or by prognostic factors, the lowest PaO_2_/FiO_2_ ratio (the ratio of arterial oxygen partial pressure to fractional inspired oxygen) documented during the hospital stay was recorded and a PaO_2_/FiO_2_ ratio < 100 was considered as a secondary criterion to identify subjects with the most severe respiratory condition. Mortality was defined as death from any cause within 90-days from hospital admission, either if occurred during hospitalization or after hospital discharge. The dates of patient’s death were gathered from the electronic mortality register.

### 2.5. Data Analysis

Based on a preliminary analysis on the first 50 enrolled patients, a prevalence of hypoalbuminemia of almost 50% was documented according to the laboratory threshold (3.5 g/dL). A minimum required sample size of 774 patients was calculated a priori assuming a prevalence of hypoalbuminemia 10% lower based on the identified new risk thresholds. This sample size enabled a type-I probability error of 5% and a desired statistical power of 80%.

The continuous variables were displayed as medians and interquartile ranges (IQRs). Unadjusted comparisons between the groups were analyzed via nonparametric Mann-Whitney’s U test for independent samples or Kruskal-Wallis test adjusted by the Bonferroni correction for multiple pairwise comparisons, as appropriate. The nominal variables were presented as numbers and percentages and the comparisons were performed via a χ test.

The performance of SA was tested by calculating the area under the curve (AUC). Youden’s *J* statistic was used to identify the optimal cut off values; the positive and negative predictive values (PPV and NPV, respectively) with relative 95% confidence intervals (CI) were calculated as well. The so-identified SA cutoffs were tested by applying ‘time-to-event’ analysis to identify patients with longer and those with shorter intervals between hospital admission and either the start of IMV or death. Comparisons were performed by fitting multivariable Cox proportional-hazards models with forward stepwise selection, controlled for age, sex and the relevant comorbid conditions. Results were reported as hazard ratios (HRs) with relative 95% CI and *p*-values (and with the respective Kaplan-Meier curves as well). Observations were right-censored after 30- and 90-days of observation for IMV and mortality analyses, respectively (known survival). Moreover, multiple linear regression and logistic regression models were used to examine the independent association between SA levels and the hospital LOS and PaO_2_/FiO_2_ ratio < 100, respectively.

All statistical analyses were performed using the software IBM SPSS Statistics, version 24.0 (New York, NY, USA: IBM Corp.), and an alpha level of *p* < 0.05 was considered statistically significant.

## 3. Results

During the study period, a total of 864 patients diagnosed with COVID-19 pneumonia were enrolled. The main characteristics of the enrolled patients are described in Table 1. Almost a quarter of patients were obese, and more than half suffered from hypertension. Hypoalbuminemia (SA < 3.5 g/dL) was detected in 586 patients (67.8%). The interval between symptoms onset and hospitalization did not differ between patients presenting hypoalbuminemia at hospital admission or not (respectively, 7.0 days, IQR 3.0–9.0, vs. 7.0 days, IQR 3.0–10.0; *p* = 0.765).

### 3.1. Albumin and Severity of Respiratory Failure

Most patients (n = 479; 55.4%) received oxygen therapy via a Venturi mask or high-flow nasal cannula (HFNC). Non invasive ventilation (NIV) was started in 352 patients (40.7%); 41 of them (11.6%) were subsequently intubated and underwent IMV. Thirty-three (3.8%) patients started IMV upon hospital admission. In total 74 patients (8.6%) underwent IMV. A PaO_2_/FiO_2_ ratio of <100 was documented in 383 patients (44.3%).

**Figure 1:**
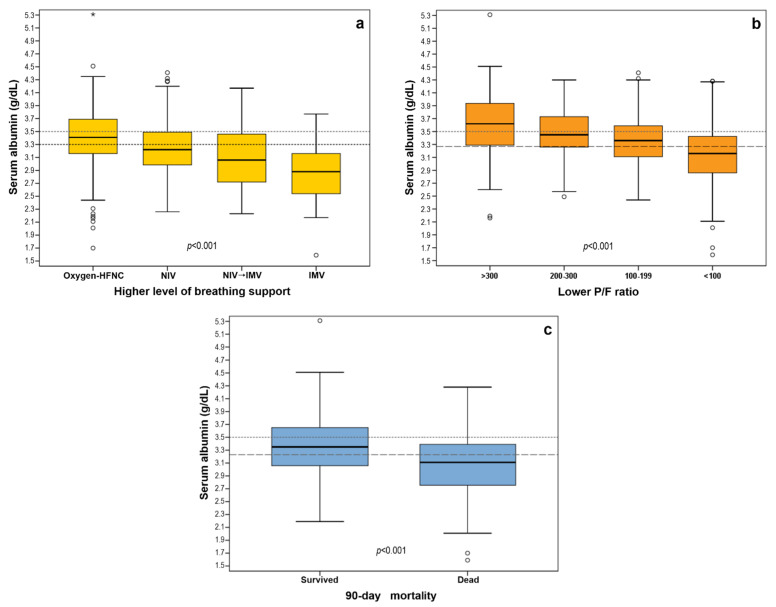
Differences among study subgroups according to serum albumin level at hospital admission: (**a**) higher level of breathing support; (**b**) lower P/F ratio; and (**c**) 90-day mortality. Black horizontal line inside the boxes: median. Boxes height: IQR. Whiskers: 1.5 × IQR. Small circles: outliers. Stars: extreme values. Small-dashed lines: serum albumin laboratory normal threshold. Large-dashed lines: serum albumin best cutoff values. HFNC: high-flow nasal cannulae. NIV: non-invasive mechanical ventilation. IMV: invasive mechanical ventilation.

In the bivariate analysis, SA on admission showed a level of decrease in parallel with the increase in the respiratory oxygen/support needed (Venturi mask → HFNC → NIV → IMV). Compared to patients receiving Venturi mask/HFNC (SA 3.4 g/dL; IQR 3.2–3.7), SA levels were significantly lower in all pairwise comparisons, being the lowest in patients who needed IMV (2.9 g/dL; IQR 2.5–3.2) (Figure 1a).

**Figure 2:**
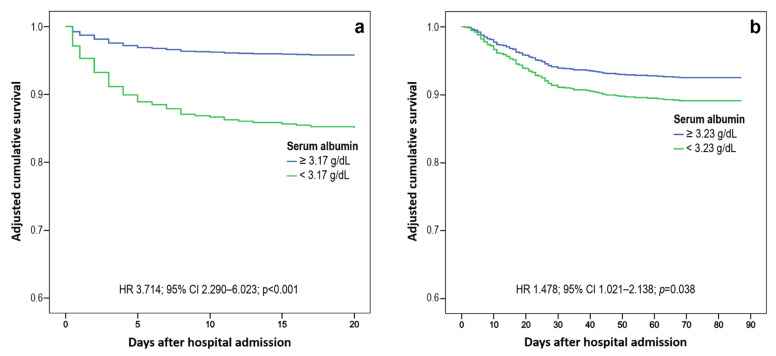
Adjusted Kaplan-Meier curves for risk to invasive mechanical ventilation (**a**) and 90-days mortality (**b**) according to the identified specific best risk thresholds of albumin at hospital admission.

The optimal SA cutoff separating patients who underwent IMV from those who did not was 3.17 g/dL (AUC 0.688; 95% CI: 0.618–0.759; *p* < 0.001), with a PPV of 15.5% (95% CI: 13.1–18.1%) and a NPV of 95.5% (95% CI: 93.9–96.7%). After applying a multivariate Cox regression analysis, SA < 3.17 g/dL at hospital admission appeared as an independent risk factor for the need of IMV (HR 3.714; 95% CI: 2.290–6.023; *p* < 0.001) (Figure 2a). Among the explored covariates, only male gender (HR 2.114, 1.180–3.786, *p* = 0.012) showed a statistically significant association with the risk of IMV in the final regression model.

SA concentration showed a statistically significant (*p <* 0.001) decrease concordantly with the categories of decreasing PaO_2_/FiO_2_ ratio. Compared to patients with a PaO_2_/FiO_2_ ratio of >300 (SA 3.6 g/dL; IQR 3.3–3.9), SA levels were significantly lower (*p* < 0.001) in all pairwise comparisons, being the lowest in patients showing a critical PaO_2_/FiO_2_ ratio of < 100 during their hospital stay (3.2 g/dL; IQR 2.9–3.4) (Figure 1b). An optimal cutoff of 3.27 g/dL was identified to identify patients at risk for a PaO_2_/FiO_2_ ratio < 100 (AUC 0.694; 95% CI 0.658–0.729; *p* < 0.001). The identified cutoff of <3.27 g/dL confirmed its independent predictive power in a multiple logistic regression model applied to ascertain the effects of the study variables on the risk of reaching a PaO_2_/FiO_2_ ratio of <100 (Table 2). 

### 3.2. Albumin and Hospital LOS

Overall, the median hospital LOS was 12 days (IQR 7–22 days) and was higher (*p* = 0.043) in patients who died before hospital discharge (n = 122; 16.0 days; IQR 8.0–25.5) as compared to those who were discharged alive (n = 775; 12.0 days; IQR 7.0–21.0). In a multiple linear regression model devised to determine the explanatory factors for the hospital LOS, a higher SA level appeared to be predictive of a shorter hospital LOS (*β* = −0.210; *p* < 0.001) (Table 3). Similar results were obtained after excluding subjects deceased before hospital discharge (*β* = −0.218; *p* < 0.001).

### 3.3. Albumin and 90-Day Mortality

At 90-day follow-up, the overall mortality rate was 14.9% (n = 134). SA was significantly lower (*p* < 0.001) in patients who had died by day 90 from hospital admission (3.1 g/dL; IQR 2.8–3.4) as compared to those who survived (3.4 g/dL; IQR 3.1–3.7) (Figure 1c). The optimal SA cutoff that separates patients deceased from those who survived within 90 days was 3.23 g/dL (AUC 0.678; 95% CI: 0.629–0.727; *p* < 0.001), with a PPV of 22.2% (95% CI: 19.5–25.2%), and a NPV of 90.4% (95% CI: 88.3–92.2%). In a multivariate Cox regression analysis, a SA of <3.23 g/dL appeared to be an independent risk factor for 90-day mortality (HR 1.478; 95% CI: 1.021–2.138; *p* = 0.038) (Figure 2b). In the final regression model, also age > 65 years (HR 6.396; 95% CI: 3.508–11.663; *p* < 0.001), metastatic cancer (HR 2.816; 1.356–5.850; *p* = 0.005), heart disease (HR 1.638; 95% CI: 1.132–2.370; *p* = 0.009), and hypertension (HR 1.550; 95% CI: 1.018–2.361; *p* = 0.041) showed a statistically significant association with 90-day mortality.

## 4. Discussion

Our study confirms that hypoalbuminemia is strongly associated with poor outcomes in hospitalized COVID-19 patients with pneumonia. In the study population, hypoalbuminemia at hospital admission was found predictive for higher risk of IMV, longer hospital LOS, and higher 90-day mortality rate.

The purpose of our study was to identify different SA thresholds in predicting the risk of severe respiratory failure, a longer hospital stay, and the risk of death in hospitalized adult patients with COVID-19 pneumonia. Qian et al. found out that 2.45 g/dL was an optimal cut off value to define hypoalbuminemia and to predict outcomes in patients with septic shock [14]. To the best of our knowledge, a universal and well-recognized cut off for hypoalbuminemia is lacking. One of the main limitations of the recently published meta-analysis about hypoalbuminemia in COVID-19 is the high variability of values used for defining low SA concentration [12], preventing subgroup analysis based on the cut off value. The added value of our study consists in the identification of thresholds for established outcomes, in order to obtain a higher sensitivity for SA levels. We identified specific SA thresholds for both the risk of severe respiratory failure (3.17 g/dL) and for 90-day risk of death (3.23 g/dL) with high NPV (96% and 90%, respectively). This means that subjects presenting with SA exceeding these thresholds at hospital admission had a low risk of experiencing severe respiratory failure during hospital stay (<4%) and of dying within 90 days (<10%).

In multivariate analyses, the risk of undergoing IMV or to die by 90 days was 3.7 and 1.5 times higher, respectively, for patients with SA below the identified threshold compared to subjects with higher SA levels, independently from age, sex, and relevant comorbidities. Predictably, when considering the identified and more specific risk thresholds, the prevalence of hypoalbuminemia on admission dropped from 68%—based on a 3.5 cut off—to 36–41%. These findings may have relevant implications in daily clinical practice and can be helpful to establish clinical priorities, intensity of care and resources allocation.

The underlying mechanisms by which low SA correlates with poor outcomes in COVID-19 are not fully understood. However, various considerations can be noted. From a pathogenetic point of view, endothelial dysfunction plays an important role in COVID-19 [15]. Endothelial dysfunction is the result of several mechanisms, including reaction to SARS-CoV-2, as well as to hypoxia, activation and recruitment of immune cells with the production of inflammatory mediators. This process leads to the loss of integrity of the epithelial–endothelial barrier, to the passage of fluids and proteins, including SA, from the intravascular to the extravascular compartments, contributing to hypoalbuminemia. Therefore, hypoalbuminemia may serve as a severity marker of epithelial–endothelial damage in patients with COVID-19 [16].

Neutrophil extracellular traps (NETs) are important mediators of tissue damage in inflammatory diseases, also in COVID-19 [17]. SA is known to inhibit NETs formation [18] and this may contribute to explain why patients with hypoalbuminemia have a higher risk of severe respiratory failure and death.

Moreover, it is important to mention the anticoagulant action of SA [19]. Low SA levels might, indeed, contribute to thrombotic events, also in COVID-19 patients, where a procoagulant inflammatory activation exists [11]. Menichelli et al. conducted a meta-regression analysis that confirmed an association with COVID-19 severity and elevated D-Dimer and low SA [20].

We assessed SA only at hospital admission. Only a few studies measured SA as well during the hospital stay. Two different retrospective observational studies found out that SA levels decreased during hospitalization. Both demonstrated that a reduction of SA concentration could be a predictor for death or progression in COVID-19 patients with hypoalbuminemia [21,22], whereas other studies observed that the normalization of SA may be also associated with a good prognosis [23]. Ali et al. demonstrated that hypoalbuminemia may persist post recovery in patients with a history of severe COVID-19 [24]. These studies confirm the prognostic role on SA in COVID-19.

Our work has some limitations that deserve to be considered when interpreting the results. Firstly, it is a monocentric observational study, and our findings cannot be generalized. Secondly, we assessed SA levels at hospital admission, regardless of when the symptoms started. Thirdly, other co-infections, also associated with hypoalbuminemia, were not adjusted for in this study, due to the lack of complete data.

The relationship between low SA levels and poor outcomes may have a strong pathophysiologic rationale. Our study demonstrates that low SA at hospital admission may identify patients with COVID-19 pneumonia at higher risk of severe respiratory failure, death, and longer LOS.

## 5. Conclusions

Our study confirms that hypoalbuminemia at hospital admission may identify patients with COVID-19 pneumonia at higher risk of severe respiratory failure, death, and longer LOS. SA level of 3.2 g/dL was identified as the better cut off value to recognize patients at risk for severe respiratory failure (IMV) and 90-day mortality.

## Figures and Tables

**Table 1:** Main characteristics of the study population at hospital admission.

Sex (n, male)	584 (67.6%)
Age (years)	66.0; 55.0–76.5
Body Mass Index	26.1; 23.9–29.4
<20 kg/m^2^	32 (3.7%)
20–29 kg/m^2^	633 (73.3%)
≥30 kg/m^2^	199 (23.0%)
Laboratory tests	
Total white blood cells × 10^3^/μL	7.6; 5.4–10.5
Neutrophils × 10^3^/μL	6.3; 4.2–9.1
Monocytes × 10^3^/μL	0.4; 0.3–0.6
Lymphocytes × 10^3^/μL	0.7; 0.5–1.0
Eosinophils × 10^3^/μL	0.0; 0.0–0.0
Platelets × 10^3^/μL	216.0; 164.0–288.0
Serum C-reactive protein (mg/L)	76.0; 31.0–130.0
Serum d-dimer (ng/mL FEU)	962.0; 625.0–1544.0
Serum albumin (g/dL)	3.3; 3.0–3.6
Past medical history	
Hypertension (n)	441 (51.0%)
Heart disease (n)	229 (26.5%)
Diabetes (n)	196 (22.7%)
Metastatic cancer (n)	15 (1.7%)

**Table 2:** Logistic regression analysis on independent predictor for PaO_2_/FiO_2_ < 100.

Predictor	OR (95% CI); *p*-Value
Serum albumin < 3.27 g/dL	3.013 (2.237–4.058); <0.001
Age > 65 years	1.730 (1.265–2.365); 0.001
BMI ≥ 30 kg/m^2^	1.688 (1.188–2.400); 0.003
Hypertension	1.504 (1.105–2.046); 0.009
Diabetes	1.502 (1.058–2.132); 0.023

OR, odds ratio; CI, confidence interval. Predictors excluded by the final model: sex, heart disease, and metastatic cancer.

**Table 3:** Multiple linear regression analysis on independent predictors of hospital length of stay.

Predictor	*β*-Value; *p*-Value
Serum albumin (g/dL)	−0.210; <0.001
Diabetes	0.127; <0.001
Hypertension	0.113; 0.001
Age > 65 years	0.098; 0.006

Predictors excluded by the final model: sex, BMI ≥ 30 kg/m^2^, heart disease, and metastatic cancer.

## Data Availability

Electronics protected datasets.

## References

[1] WHO Coronavirus Disease (COVID-19). www.covid19.who.int.

[2] Hu B., Guo H., Zhou P., Shi Z.-L. (2020). Characteristics of SARS-CoV-2 and COVID-19. Nat. Rev. Microbiol..

[3] Sambataro G., Giuffrè M., Sambataro D., Palermo A., Vignigni G., Cesareo R., Crimi N., Torrisi S.E., Vancheri C., Malatino L. (2020). The Model for Early COvid-19 Recognition (MECOR) Score: A Proof-of-Concept for a Simple and Low-Cost Tool to Recognize a Possible Viral Etiology in Community-Acquired Pneumonia Patients during COVID-19 Outbreak. Diagnostics.

[4] Yin M., Si L., Qin W., Li C., Zhang J., Yang H., Han H., Zhang F., Ding S., Zhou M. (2018). Predictive Value of Serum Albumin Level for the Prognosis of Severe Sepsis Without Exogenous Human Albumin Administration: A Prospective Cohort Study. J. Intensive Care Med..

[5] Sung J., Bochicchio G.V., Joshi M., Bochicchio K., Costas A., Tracy K., Scalea T.M. (2004). Admission Serum Albumin Is Predictive of Outcome in Critically Ill Trauma Patients. Am. Surg..

[6] Fanali G., di Masi A., Trezza V., Marino M., Fasano M., Ascenzi P. (2012). Human Serum Albumin: From Bench to Bedside. Mol. Aspects Med..

[7] Caironi P., Gattinoni L. (2009). The Clinical Use of Albumin: The Point of View of a Specialist in Intensive Care. Blood Transfus..

[8] Gounden V., Vashisht R., Jialal I. (2021). Hypoalbuminemia. StatPearls [Internet].

[9] Wiedermann C.J. (2021). Hypoalbuminemia as Surrogate and Culprit of Infections. Int. J. Mol. Sci..

[10] Huang J., Cheng A., Kumar R., Fang Y., Chen G., Zhu Y., Lin S. (2020). Hypoalbuminemia Predicts the Outcome of COVID-19 Independent of Age and Co-Morbidity. J. Med. Virol..

[11] Kheir M., Saleem F., Wang C., Mann A., Chua J. (2021). Higher Albumin Levels on Admission Predict Better Prognosis in Patients with Confirmed COVID-19. PLoS ONE.

[12] Soetedjo N.N.M., Iryaningrum M.R., Damara F.A., Permadhi I., Sutanto L.B., Hartono H., Rasyid H. (2021). Prognostic Properties of Hypoalbuminemia in COVID-19 Patients: A Systematic Review and Diagnostic Meta-Analysis. Clin. Nutr. ESPEN.

[13] Dessie Z.G., Zewotir T. (2021). Mortality-Related Risk Factors of COVID-19: A Systematic Review and Meta-Analysis of 42 Studies and 423,117 Patients. BMC Infect. Dis..

[14] Qian S., Jin D., Chen Z., Ye Y., Xiang W., Ye L., Pan J.-Y. (2019). Hypoalbuminemia, a Novel Prognostic Factor for Prediction of Long-Term Outcomes in Critically Ill Patients with Septic Shock. Int. J. Clin. Exp. Med..

[15] Bakker J., Horowitz J.M., Hagedorn J., Kozloff S., Kaufman D., Castro R. (2021). Blood Volume and Albumin Transudation in Critically Ill COVID-19 Patients. Crit. Care.

[16] Wu M.A., Fossali T., Pandolfi L., Carsana L., Ottolina D., Frangipane V., Rech R., Tosoni A., Lopez G., Agarossi A. (2021). Hypoalbuminemia in COVID-19: Assessing the Hypothesis for Underlying Pulmonary Capillary Leakage. J. Intern. Med..

[17] Veras F.P., Pontelli M.C., Silva C.M., Toller-Kawahisa J.E., de Lima M., Nascimento D.C., Schneider A.H., Caetité D., Tavares L.A., Paiva I.M. (2020). SARS-CoV-2-Triggered Neutrophil Extracellular Traps Mediate COVID-19 Pathology. J. Exp. Med..

[18] Neubert E., Senger-Sander S.N., Manzke V.S., Busse J., Polo E., Scheidmann S.E.F., Schön M.P., Kruss S., Erpenbeck L. (2019). Serum and Serum Albumin Inhibit in Vitro Formation of Neutrophil Extracellular Traps (NETs). Front. Immunol..

[19] Paar M., Rossmann C., Nusshold C., Wagner T., Schlagenhauf A., Leschnik B., Oettl K., Koestenberger M., Cvirn G., Hallström S. (2017). Anticoagulant Action of Low, Physiologic, and High Albumin Levels in Whole Blood. PLoS ONE.

[20] Menichelli D., Di Rocco A., del Sole F., Pignatelli P., Vestri A., Violi F., Pastori D. (2021). Serum Albumin, Clotting Activation and COVID-19 Severity: A Systematic Review and Meta-Regression Analysis of 4579 Patients. Ital. J. Emerg Med..

[21] Van Zyl J.S., Alam A., Felius J., Youssef R.M., Bhakta D., Jack C., Jamil A.K., Hall S.A., Klintmalm G.B., Spak C.W. (2021). ALLY in Fighting COVID-19: Magnitude of Albumin Decline and Lymphopenia (ALLY) Predict Progression to Critical Disease. J. Investig. Med..

[22] Feng R., Wang B., Ma Z., Guo X., Li H., Tang Y., Meng H., Yu H., Peng C., Chu G. (2021). Dynamic Change of Serum Albumin Level Can Predict the Prognosis of COVID-19 Patients with Hypoalbuminemia. J. Med. Virol..

[23] Su C., Hoffman K.L., Xu Z., Sanchez E., Siempos I.I., Harrington J.S., Racanelli A.C., Plataki M., Wang F., Schenck E.J. (2021). Evaluation of Albumin Kinetics in Critically Ill Patients With Coronavirus Disease 2019 Compared to Those With Sepsis-Induced Acute Respiratory Distress Syndrome. Crit. Care Explor..

[24] Ali K.M., Ali A.M., Tawfeeq H.M., Figueredo G.P., Rostam H.M. (2021). Hypoalbuminemia in Patients Following Their Recovery from Severe Coronavirus Disease 2019. J. Med. Virol..

